# Reproducibility and construct validity of the utrecht mixing ability Test to obtain masticatory performance outcome in patients with condylar mandibular fractures

**DOI:** 10.1111/joor.12917

**Published:** 2019-12-11

**Authors:** Florine M. Weinberg, Jorine A. Vermaire, Tymour Forouzanfar, Antoine J.W.P. Rosenberg, Caroline M. Speksnijder

**Affiliations:** ^1^ Department of Oral and Maxillofacial Surgery and Special Dental Care University Medical Center Utrecht Utrecht University Utrecht The Netherlands; ^2^ Division of Medical Imaging Department of Radiation Oncology University Medical Center Utrecht Utrecht University Utrecht The Netherlands; ^3^ Department of Oral and Maxillofacial Surgery/Pathology Amsterdam UMC Vrije Universiteit Amsterdam and Academic Center for Dentistry Amsterdam (ACTA) Amsterdam The Netherlands

**Keywords:** chewing, mandibular condyle, mandibular fracture, mixing ability test, reproducibility

## Abstract

**Objective:**

This study assessed the test‐retest reproducibility of the Utrecht mixing ability test (MAT) and the construct validity of the MAT in relation to the Mandibular Function Impairment Questionnaire (MFIQ) in patients with mandibular condylar fractures.

**Material and methods:**

Twenty‐six patients treated for a mandibular condylar fracture participated in this clinimetric study; all patients performed the MAT twice. Simultaneously the MFIQ was conducted. Test‐retest reliability and construct validity were assessed using the intra‐class correlation coefficient (ICC) and Spearman correlation, respectively.

**Results:**

The ICC of the MAT was 0.906 (95% CI: 0.801‐0.957), which indicates an excellent reliability. A weak correlation of 0.386 (*P* = .052) between the first MAT and the overall outcome of the MFIQ was found. A significant moderate correlation of 0.401 (*P* = .042) was found between the retest of the MAT and the overall outcome of the MFIQ. One question on the MFIQ (about yawning) showed a moderate positive correlation of 0.569 (*P* = .002) and 0.416 (*P* = .034) for the MAT test and retest, respectively.

**Conclusion:**

The MAT is an easy test to use in follow‐up of patients. The test‐retest reliability of this test is excellent in condylar trauma patients. As the validity of the MAT and the MFIQ could not be confirmed, the MFIQ may be an addition to patient's feedback about the rehabilitation process of their mandibular functioning.

## 1 INTRODUCTION

After a maxillofacial injury, patients frequently have problems with eating food (67%) and have to change their diet (55%) due to decreased masticatory functioning.^1^ Mastication is a complex cooperation of different mechanical and chemical mechanisms. Mobility of the temporomandibular joint, facial musculature, bite and tongue force, sensory relations, occlusal units and saliva production all play a role in mastication.^2^ The mandible is fractured in 36% to 54% of all patients with maxillofacial trauma.^3^, ^4^ In 36% to 44% of mandibular fractures, the mandibular condyle is involved.^3^, ^4^ Such a fracture can influence masticatory functioning due to anatomical change to the mandible or injury to the nerve or musculature.^5^, ^6^ However, the therapy received may influence mastication by complications due to open reduction and internal fixation, such as fistulas of the parotid gland and/or facial nerve damage or hardware problems. Similarly, ankyloses of the temporomandibular joint or limited mouth opening can occur due to long‐term immobilisation in the case of conservative treatment.^7^ Thereby, malocclusion, limited range of motion of the mouth or chronic pain can disturb the mastication process.^8^

Masticatory performance is the objective efficiency of this mastication process, which can be measured by different methods (*fi* comminution or mixing ability methods).^9^ The Utrecht mixing ability test (MAT) with two‐coloured wax was described as a reliable test for patients with cerebral palsy syndrome.^10^ For patients with mandibular trauma, such as condylar fracture, the reproducibility and validity of this test have not yet been investigated.^2^, ^9^

Masticatory ability is the subjective testing of the mastication process, which reflects the expectations of the patients and their quality of life by taking the psychological and emotional adjustment of the patient in their daily life into account. This can be an advantage over measurement of objective outcomes alone. Subjective efficiency of the mastication process is tested in various departments with several questionnaires, such as the Oral Health Impact Profile‐14 questionnaire (OHIP‐14)^11^ and the Mandibular Function Impairment Questionnaire (MFIQ).^12^, ^13^ In recent studies, the MFIQ has been used to measure subjective masticatory ability in condylar trauma patients.^5^, ^14^

It is important to the rehabilitation of the patient to get insight into mastication after mandibular injury by performing reliable and valid tests.^15^ Therefore, the aim of this study is to determine, on the one hand, the test‐retest reproducibility (reliability, measurement error and agreement) of the MAT, and, on the other hand, the construct validity of the MAT in relation to the MFIQ in patients with mandibular condylar fractures. We hypothesise that the reproducibility of the MAT will be sufficient (ICC ≥ 0.7) and that the construct validity would be at least moderately correlated (≥ 0.60).

## 2 MATERIAL AND METHODS

### 2.1 Subjects

Patients treated for a mandibular condylar fracture at the Department of Oral and Maxillofacial Surgery of the University Medical Center Utrecht (UMCU), Amsterdam UMC, Vrije Universiteit Amsterdam and Onze Lieve Vrouwe Gasthuis (OLVG) Amsterdam between June 2017 and January 2019 were recruited for this study. Inclusion criteria were as follows: (a) 18 years or older; (b) condylar base or neck fracture, with or without additional fracture locations of the mandible; (c) presence of disocclusion; and (d) dislocation of the fracture, caused by trauma. Exclusion criteria were as follows: (a) additional midface fracture; (b) legal incapability; (c) inoperable conditions because of comorbidity; and (d) inability to understand the Dutch language. Sex and age were retrieved from clinical records.

All patients had to be stable on the interim period of measurements, and the test conditions and test instructions were kept similar for all subjects.

Study power was calculated based on sample size calculation for reliability studies by Walter et al^16^ An ICC of at least 0.7 (ρ_0_ = 0.7 and ρ_1_ = 0.9) showed a sample size of 18.4 patients.^16^ A second power analysis based on the MAT reproducibility and validity in a comparable study in children with cerebral palsy showed a sample size of 25‐30 patients.^10^ Therefore, we choose to include 26 patients.

The study protocol was approved by the Ethics Committee of UMC Utrecht (NL59658.041.16). All subjects received a written explanation of the study, and informed consent was obtained from each subject before the start of the tests.

### 2.2 Masticatory performance

A comprehensive description of the mixing ability test (MAT) as developed by the University Medical Center Utrecht was published previously.^2^, ^17^, ^18^ The MAT quantifies how well a patient is able to mix two layers of red and blue colour of a wax tablet by chewing a certain number of strokes, which are digitally analysed afterwards. The outcome variable is called the mixing ability index (MAI) and ranges between 5 and 30, where a score of 5 means a fully mixed tablet and 30 an unused wax tablet. A lower MAI implies a better mixed tablet, hence a better masticatory performance.

The tablet consists of two 3‐mm thick layers of coloured Plasticine modelling wax (non‐toxic DIN EN‐71, art. nos. crimson 52 801 and blue 52 809, Stockmar) with a diameter of 20 mm. It is used at room temperature (20°C) and forms a compact bolus during chewing. Each subject was instructed to chew 15 times on the tablet.

A repetition of 15 times was chosen for this trauma group because the authors assume that this group has no problems with tongue mobility or dentition, in contrast to oncological patients, for whom this test was originally designed.^2^ A ceiling in outcome will be received when chewing more strokes.^2^ This procedure was repeated with a second wax tablet, with an appropriate time interval of 15 minutes minimum. Thereafter, the chewed tablets were removed, flattened between foil to a thickness of 2.0 mm and photographed on both sides using a high‐quality scanner (Epson V750, Long Beach). The retrieved images were analysed and processed using Adobe Photoshop, CS3 extended (Adobe, San Jose), a commercially available program for image analysis. The MAI was obtained by measuring the intensity distributions of the red and blue colouring on the combined image on both sides of the flattened wax.

### 2.3 Mandibular function impairment questionnaire

The Mandibular Function Impairment Questionnaire (MFIQ) is designed to assess the masticatory ability, or, in other words, the patient's perception of mandibular function impairment. The MFIQ has been proven reliable in patients with painfully restricted temporomandibular joints by a moderate to good test‐retest reliability (Spearman correlation of 0.69 to 0.96).^13^

The minimal amount of change to be detected is 14 units on a scale of 0 to 68,^13^ where 0 indicates no mandibular function impairment and 68 a poor functional outcome. The MFIQ consists of 17 items. Each item is presented with a 5‐point Likert scale, on which the patient can indicate how much difficulty was experienced while performing a particular mandibular movement or task (eg speech, daily activities, drinking, laughing, yawning and eating different types of food). The scores are as follows: 0 = no difficulty, 1 = a little difficulty, 2 = quite a bit of difficulty, 3 = much difficulty and 4 = very difficult or impossible without help.

### 2.4 Statistical analysis

#### 2.4.1 Reproducibility of the test‐retest

The test‐retest reproducibility is divided into reliability and agreement parameters. Reliability (the proportion of the total variance in the measurements that is due to “true” differences among patients) of the MAT was calculated with an intra‐class correlation coefficient (ICC) with corresponding 95% confident intervals, based on a mean rating (*k* = 2), absolute agreement, two‐way random‐effects model and single measures (ICC 2.1). This is calculated as: (*MS_R_ ‐ MS_E_) / (MS_R_ + (k ‐ 1)MS_E_ + (k / n) (MS_C_ ‐ MS_E_*)), with *MS_R_* = mean square for rows; MS_E_ = mean square for error; *MS_C_* = mean square for columns; and k = number of raters/measurements. Cut‐off points for the ICC were chosen as <0.5 = poor, 0.5 to 0.75 = moderate, 0.75 to 0.90 = good and >0.90 = excellent reliability.^19^ A threshold of 0.75 for the ICC was taken as an acceptable level of test‐retest reliability.^19^

The measurement error consists of the systematic and random error of a patient's score, which is not attributed to true changes in the construct of disability. Agreement was assessed by calculating the standard error of measurements (SEM) of the MAT. The SEM is a measure of how much measured test scores are spread around a “true” score. This is calculated from the ICC as *SEM_agreement_ = *SD * √(1 ‐ ICC), with SD meaning “standard deviation of the differences of the MAT.” The SEM_agreement_ was additionally used to calculate the smallest detectable change values at the individual level (SDC_ind_), using the equation *1.96* *× √2 × SEM_agreement_*, to yield 95% confidence that the observed change was real and not attributable to the measurement error. Limits of agreement (LoA) estimate the interval at which a proportion of the differences between measurements is positioned. These were calculated as upper LoA = mean +1.96 * SD and lower LoA = mean ‐1.96 * SD. The Bland‐Altman plot was constructed to provide a visual representation of the presence of systematic errors. The Bland‐Altman plot was based around three variables: the mean systematic difference between test and retest scores and the upper and lower limits of agreement, which span 95% of observations, assuming that the values for the difference between test and retest scores are distributed normally. These variables were integrated into a scatter plot where the difference between test and retest values was put on the y‐axis, and the average of the test and retest values was put on the x‐axis.^19^, ^20^

#### 2.4.2 Construct validity

Construct validity was determined by hypothesis testing using Spearman's correlation. It was hypothesised that mastication assessed by the MAT and MFIQ (per item and summary score) would be at least moderately correlated (≥0.60). Cut‐off points for the validity were chosen as: 0.00 to 0.19 = very weak, 0.20 to 0.39 = weak, 0.40 to 0.59 = moderate, 0.60 to 0.79 = strong and 0.80 to 1.00 = very strong.^21^

A *P*‐value of less than 0.05 was considered statistically significant. A Spearman's correlation was run to determine the relationship between the MAT and the outcomes of the MFIQ. All analyses were performed using SPSS version 25 (IBM Corporation).

## 3 RESULTS

Twenty‐six patients were included in this study and are depicted in Table 1. Eighteen patients (69%) were male, and the mean age was 41 years with a range of 18 to 69. Twenty‐two subjects underwent the mixing ability test and retest six weeks after treatment for condylar fracture, and the remaining four subjects underwent the test and retest after six months.

**Table 1:** Participants’ characteristics and outcomes

	Total patient group n = 26
Sex	
Male, n (%)	18 (69)
Female, n (%)	8 (31)
Age (years), mean (Range)	41 (18‐69)
Fracture type	
Condylar neck fracture, n (%)	7 (27)
Condylar base fracture, n (%)	19 (73)
Hospital	
UMC Utrecht, n (%)	15 (58)
Amsterdam UMC, VUmc, n (%)	1 (4)
OLVG, n (%)	10 (38)
Treatment of the fracture	
Operative, n (%)	16 (62)
Conservative (MMF), n (%)	10 (38)
Timing of test‐retest	
6 weeks post‐treatment, n (%)	22 (85)
6 months post‐treatment, n (%)	4 (15)

Abbreviations: Amsterdam UMC, VUmc: Amsterdam University Medical Centra, Vrije Universiteit Medical center; MMF: Maxillomandibular Fixation; OLVG: Onze Lieve Vrouwe Gasthuis, Amsterdam; UMC Utrecht: University Medical Center Utrecht.

### 3.1 Reproducibility of the test‐retest

**Figure 1:**
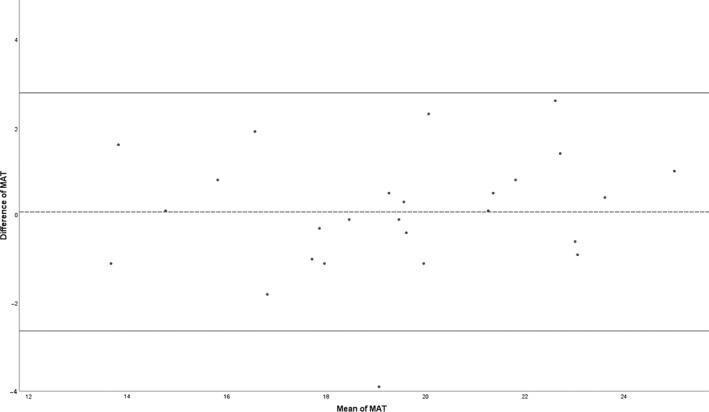
Bland‐Altman plot for the test‐retest reproducibility of the Mixing Ability Test. The dashed line represents the mean difference, and the solid lines represent the 95% limits of agreement. MAT: Mixing Ability Test

The MAI of the test had a mean of 19.44 (SD: 3.23). The MAI of the retest had a mean of 19.37 (SD: 3.02). In this condylar trauma patient group, the ICC of the MAT was 0.906 (95% CI: 0.801‐0.957), which means an excellent reliability. The results of the SD, SEM, SDC and a Bland‐Altman plot with corresponding LoA can be found in Table 2 and Figure 1. Systemic bias was visually assessed by Bland‐Altman. It showed a consistent variability across the graph.

**Table 2:** Test‐retest reproducibility

Results	
MAI	
‐ Test, mean (SD)	19.44 (3.23)
‐ Retest, mean (SD)	19.37 (3.02)
Difference Test‐Retest, mean (SD)	0.07 (1.38)
ICC, (95% CI)	0.906 (0.801‐0.957)
SEM*_agreement_*	0.43
SDC	1.19
95% LoA	‐2.632 to 2.778

Abbreviations: CI: confidence interval; ICC: intra‐class correlation coefficient; LoA: limits of agreement; MAI: mixing ability index; SD: standard deviation; SDC: smallest detectable change; SEM*_agreement_*: standard error of measurement.

### 3.2 Construct validity

At the first measurement moment, there was a weak‐positive correlation of 0.386 between the MAT and MFIQ, which was not significant (*P* = .052). A significant moderate correlation of 0.401 (*P* = .042) was found between the overall outcome of the MFIQ and the retest of the MAT. One question on the MFIQ (about yawning) showed a moderate positive correlation of 0.569 (*P* = .002) and 0.416 (*P* = .034) for the MAT test and retest, respectively (see also Table 3).

**Table 3:** Correlation MAT and MFIQ

MFIQ Question	Question topic	r‐1 Test	*P*‐value	r‐2 Retest	*P*‐value
1	Social activities	‐0.022	.916	0.103	.617
2	Speaking	0.023	.913	0.130	.526
3	Biting	0.354	.076	0.425	.030
4	Hard food	0.338	.092	0.299	.138
5	Soft food	0.119	.562	0.234	.250
6	Daily activities	0.301	.135	0.432	.027*
7	Drinking	0.003	.988	0.226	.267
8	Laughing	0.169	.409	0.320	.111
9	Chewy food	0.314	.119	0.278	.170
10	Yawning	0.569	.002**	0.416	.034*
11	Kissing	0.244	.230	0.172	.401
12	Hard cookies	0.237	.244	0.228	.263
13	Meat	0.299	.138	0.234	.250
14	Raw carrot	0.381	.055	0.314	.118
15	Baguette	0.286	.156	0.252	.214
16	Nuts	0.267	.187	0.254	.211
17	Whole apple	0.191	.375	0.125	.542
Total MFIQ outcome		0.386	.052	0.401	.042*

Abbreviations: MAT: Mixing Ability Test; MFIQ: Mandibular Function Impairment Questionnaire.

* Correlation is significant at the 0.05 level (2‐tailed).

** Correlation is significant at the 0.01 level (2‐tailed).

## 4 DISCUSSION

This study focused on the test‐retest reproducibility (reliability, measurement error and agreement) and construct validity of the MAT in patients with mandibular condylar fractures. We found an excellent reliability of the Utrecht mixing ability test in patients with a condylar fracture of the mandible. The SEM is 0.43, which is very small considering the range of outcome possibilities of the MAT. The SDC for the MAT in this group of condylar trauma patients is 1.19. This means that the MAI of an individual would have to change by at least 1.19 points before the observed change can be considered to be a true change in the masticatory performance of a subject and not potentially the result of measurement error. The limits of agreement are clinically interpreted as narrow. The Bland‐Altman analysis visually showed that 95% of all data lies between the upper and lower LoA, with a consistent variability. These findings are acceptable.

As hypothesised, the ICC of 0.903 indicated an excellent test‐retest reliability in patients with condylar fractures. In comparison, the ICC of the same MAT in children with cerebral palsy and typical development is 0.69.^10^

The hypothesis that the outcome of the MAT and MFIQ is at least moderately correlated could not be confirmed since the weak‐positive correlation (r = 0.39) of the first test was not convincingly significant (*P* = .052). There was a moderate correlation of the retest with the MFIQ (r = 0.40) that was significant (*P* = .042). This weak‐moderate correlation could possibly be explained by the fact that the MFIQ reflects the subjective masticatory ability and the MAT the objective masticatory performance. The MFIQ also comprehends questions about other aspects of the mandible whereas the MAT reflects the outcome of the complex masticatory process of oral muscle movements and coordination.

Nonetheless, in a normal follow‐up situation the patient will only undergo the MAT once, the test in this study, with a mean of 19.44 (Range 13.1‐25.5, SD 3.23). The retest had a mean of 19.37 (Range 13.0‐24.5, SD 3.02), which was a bit better and more consistent. This could be an explanation for the small difference in significance of the correlation between the MFIQ with the test and the MFIQ with the retest. In a cross‐sectional study, they also found a significant but weak‐positive correlation between MAT and MFIQ in patients with condylar fractures (r = 0.25 with *P* = .033).^5^ However, as also seen in other medical fields, objective functionality does not necessarily correspond to subjective quality‐of‐life outcomes for patients.^22^, ^23^, ^24^ These studies concluded that combining measurements of objective functioning and subjective functioning (in this study the MAT and MFIQ) are complementary to each other, should strengthen each other and lead to treatment in a way that meets the needs of patients.^24^

Mandibular condylar fractures often go along with disc displacement of the temporomandibular joint.^15^ Disruption in the anatomy of this joint interferes with its physiology and therefore affects the maximum mouth opening. This could be an explanation for the positive significant correlation between the question about yawning and the MAT, since yawning requires a large mouth opening movement. This is consistent with findings in another study with patients treated for oral cancer, where the authors concluded that MMO significantly contributed to the MAI. 18

The authors expected the retest to generate a better outcome than the first test for two reasons. The first reason was that when a patient had just finished treatment of the condylar fracture, and the first thing the patient was allowed to chew on was the MAT tablet, the patient's chewing performance might be limited by fear (of *f.i.* pain), also known as kinesiophobia. Our second idea involved the presence of a learning curve in the method of chewing the wax tablet. As seen in Table 1, this difference in outcome is limited to a minimum. This minimal difference could be explained by possible fatigue of the masticatory muscles when taking the retest, despite the set time between taking the retest.

### 4.1 Strengths and limitations

All results were written down according to the COnsensus‐based Standards for the selection of health Measurement INstruments (COSMIN) to ensure methodological quality.^25^ The data of this study were collected with a prospective design. All data were collected by the same author (FMW). The MATs were evaluated by the same observer (CMS).

In general, this study was conducted with a fair sample size, with two different follow‐up periods. The participants came from three different hospitals, resulting in a heterogeneous sample. One limitation of this study is that measurements on inter‐rater reliability are missing. An additional measurement was judged to be too time‐consuming for participants. In a usual care or research setting, most evaluative measurements would be performed by the same person.

As the subjects in this study were patients with condylar trauma, we have to be careful to generalise these results to general oral and maxillofacial trauma patients, and, in particular, all mandibular traumas.

### 4.2 Future research

As the treatment modality of patients with condylar trauma is still subject to debate, investigations like the MAT and MFIQ could help determine whether open surgery is preferable to conservative treatment, or vice versa. Based on the results of this study, we expect the outcome of the MAT to be of excellent reliability, and therefore, reliable conclusions can be made.

### 4.3 Conclusion

The test‐retest reliability of the MAT is excellent in condylar trauma patients and may be used in follow‐up in prospective studies. As the validity of the MAT and the MFIQ is not convincing, the MFIQ could be an addition to patient feedback about the rehabilitation process of their mandibular functioning.

## CONFLICTS OF INTEREST

The authors report no financial conflicts of interest.

## ETHICAL APPROVAL

No ethical approval was needed.

## CONSENT CO‐AUTHORS

All authors have viewed and agreed to this submission.
